# Prolonged acute migraine with aura and reversible brain MRI abnormalities after liquid sclerotherapy

**DOI:** 10.1186/1129-2377-15-41

**Published:** 2014-06-19

**Authors:** Yassine Zouitina, Mathilde Terrier, Marie Hyra, Djohar Seryer, Jean-Marc Chillon, Jean-Marc Bugnicourt

**Affiliations:** 1 Department of Neurology, Amiens University Hospital, 1 Place Victor Pauchet, F-80054 Amiens cedex, France; 2 Department of Physical Medicine and Rehabilitation, Amiens University Hospital, Amiens, France; 3 Department of Radiology, Amiens University Hospital, Amiens, France; 4 INSERM U1088, Amiens, France; 5 Department of Clinical Pharmacology, Amiens University Hospital, Amiens, France; 6 Laboratory of Functional Neuroscience and Pathology (EA 4559), Department of Neurology, Amiens University Hospital, Amiens, France

**Keywords:** Migraine, Migraine aura, Sclerotherapy, Magnetic resonance imaging, Gradient-echo T2*-weighted imaging

## Abstract

Transient visual disturbances constitute the most commonly reported neurological side effect during and immediately after sclerotherapy. A few studies, based on clinical and diffusion-weighted MRI assessments, have suggested that these transient neurological symptoms correspond to migraine with aura. Recently, it has been reported that brain magnetic resonance imaging can reveal transient T2*-weighted abnormalities during the acute phase of migraine with aura. We reported a 36-year-old man who presented with transient neurological symptoms and concomitant T2*-weighted abnormalities on brain magnetic resonance imaging immediately after liquid sclerotherapy. We hypothesize that the reversible nature of the patient’s T2*-weighted abnormalities may indicate a relationship with the post-sclerotherapy migraine with aura attack.

## Background

Transient visual disturbances constitute the most commonly reported neurological side effect during and immediately after sclerotherapy, with an incidence of 1.4%s [1]. Furthermore, foam sclerotherapy appears to be associated with a higher incidence of transient visual disturbances than liquid sclerotherapy [2]. Research has suggested that these transient neurological symptoms (which are more frequent in patients with patent foramen ovale (PFO) and/or a history of migraine) correspond to migraine with aura (MA) [3-5].

More recently, it has been reported that brain magnetic resonance imaging (MRI) can reveal transient T2*-weighted abnormalities during the acute phase of MA [6-8].

Here, we report on a patient who presented with transient neurological symptoms and concomitant T2*-weighted abnormalities on MRI immediately after liquid sclerotherapy. We hypothesize that the reversible nature of the patient’s T2*-weighted abnormalities may indicate a relationship with the post-sclerotherapy MA attack.

## Case presentation

**Figure 1:**
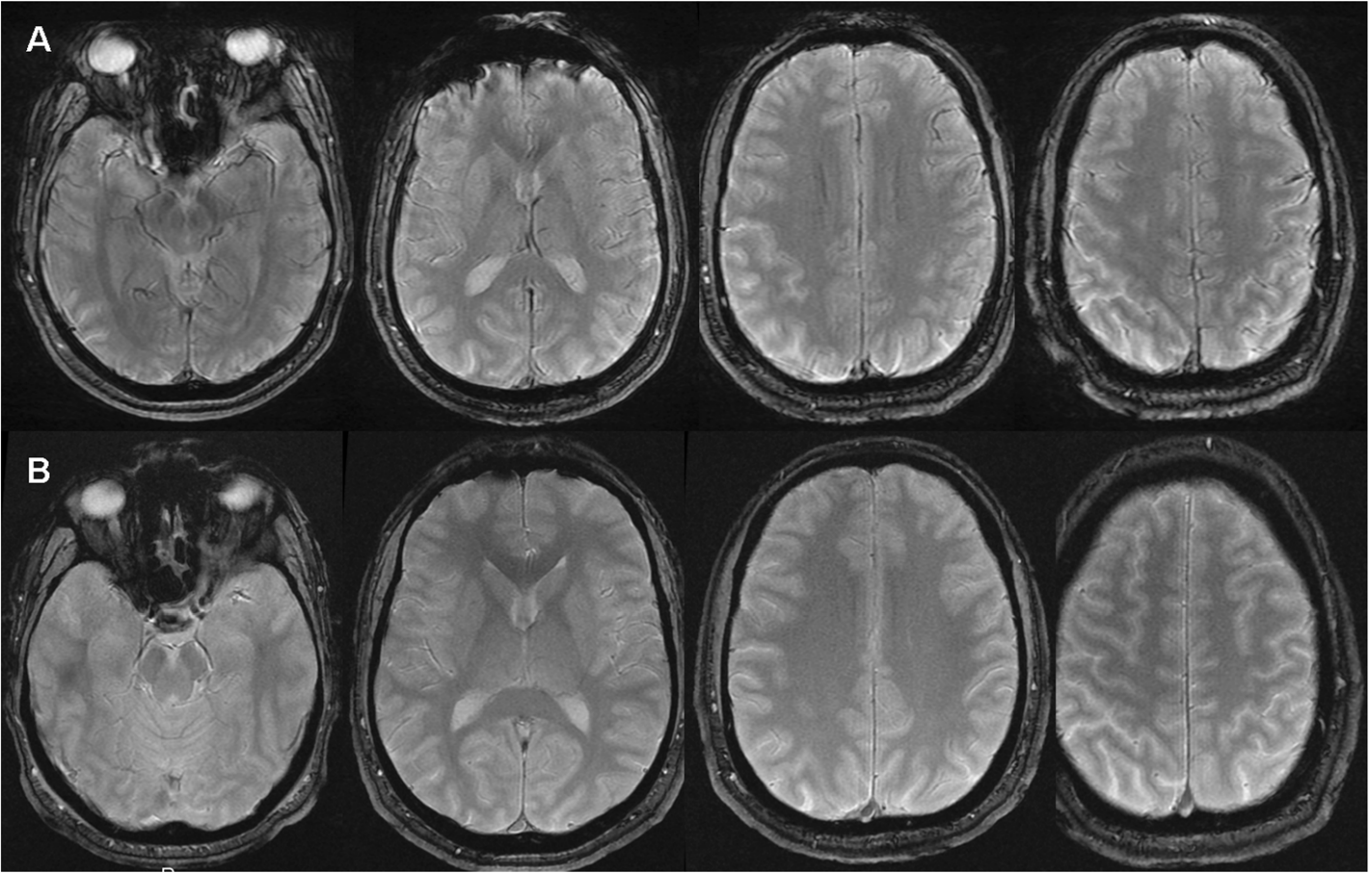
A gradient-echo T2*-weighted magnetic resonance image showing hypointense signals in both hemispheres of the brain but especially in the left hemisphere (A) and a normal gradient-echo T2*-weighted magnetic resonance image acquired five days after the migraine attack (B).

A 36-year-old man presented with symptomatic but moderate varicosity of the left small saphenous vein. The patient had no vascular risk factors, no history of venous diseases, no family history of migraine and no reported migraine comorbidities. He reported a few episodes of headache, the description of which was compatible with migraine without aura. In February 2014, he underwent liquid sclerotherapy (carried out in accordance with the European consensus statement) [9]. After contraindications to treatment were ruled out and the patient had given his written, informed consent, the first sclerotherapy session (with a total of 2 ml of 0.25% lauromacrogol solution) was not followed by any complications. Two weeks later, the patient received a second injection of 4 ml of 0.25% lauromacrogol solution. Immediately following the injection, the patient reported flickering lights in his right eye and several minutes of photopsiae, followed by right hemianopsia. These symptoms disappeared after two hours. Two weeks later, the patient underwent a third sclerotherapy (with 4 ml of 0.25% lauromacrogol solution). Immediately following injection of the liquid, the patient again reported flickering lights in his right eye, followed by right hemianopsia and (two hours later) the progressive onset of aphasia and psychomotor slowing. Comprehension was not affected. An evaluation by a neurologist revealed headache, right hemianopia, mild word-finding difficulties and a slowly progressing disturbance of consciousness. The National Institutes of Health Stroke Scale (NIHSS) score was 4 out of 42 [10]. Brain MRI (performed three hours after symptom onset) was normal. However, gradient-echo T2*-weighted images revealed several hypointense areas in both hemispheres of the brain (though predominantly in the left hemisphere) (Figure 1A). The patient’s movement prevented us from interpreting the results of magnetic resonance angiography of the Circle of Willis. Since the acute symptoms persisted, acute encephalopathy was suspected. Although the results of a cerebrospinal fluid analysis were normal, treatment with acyclovir was initiated. The chest radiography was unremarkable. The electrocardiogram, carotid ultrasonography, transcranial Doppler ultrasound and transthoracic echocardiography results were normal. The laboratory test results (including thyroid function, arterial blood gas measurement, and syphilis screening tests) were also normal. The coagulation work-up did not show any factor V Leiden or prothrombin gene G20210A mutations, and protein C, protein S, antithrombin III, factor VIII and homocysteine levels were normal. Antibody screening was negative. Transoesophageal echocardiography revealed a PFO with an associated atrial septal aneurysm. A color flow duplex scan revealed a moderate right-to-left shunt but only during provocation tests. The cardiac valves were normal, and there was no evidence of aortic atheroma or pulmonary arterial hypertension. The colour duplex ultrasonography results for the lower limbs were normal. After 48 h, the symptoms (including the headache) resolved spontaneously and the patient was diagnosed with probable migraine with aura (on the basis of this first episode of migraine with prolonged aura). Brain MRI was repeated five days after symptom onset and the T2* images were normal (Figure 1B). The patient was discharged six days after admission, with a favourable outcome (NIHSS score: 0). Following hospitalisation, the patient suffered from to other episodes of migraine with aura lasting for less than 1 hour.

## Conclusions

In migraine with aura, visual disturbances may comprise additional features (such as flickering lights and spots), or the loss of features (such as hemianopia and loss of vision) and may be associated with sensory disturbance and speech impairment (depending on the extent of cortical spreading depression (CSD) [1,11]. These complications appear to be more frequent after foam sclerotherapy than after liquid sclerotherapy [2]. Although the degree to which the injected volume of sclerotic agent contributes to the development of neurological side effects is subject to debate, it appears reasonable to decrease the volume when (i) neurological symptoms occurs after sclerotherapy and (ii) another sclerotherapy session is required [11]. Recent clinical and brain MRI studies have shown that these transient neurological symptoms (which are more frequent in patients with PFO) correspond to MA rather than to transient ischemic events [5,12].

Migraine with aura occurs in about one third of migraine sufferers [13]. It is clinically defined by at least two recurrent episodes of fully reversible symptoms (the most frequent of which are visual disturbances, sensory disturbances and speech and/or language impairment). The aura symptom spreads gradually over a period of 5 minutes and (for each individual aura) lasts between 5 and 60 minutes (although this upper limit was set arbitrarily). Indeed, it has been reported that aura lasts for more than one hour in up to 37% of patients [14]. This epidemiological reality has been recently taken into consideration in the third edition of the International Classification of Headache Disorders, in which aura lasting more than an hour but less than a week (in the absence of radiologically confirmed brain ischemia) was defined as “probable migraine with aura (prolonged aura)” [15]. These atypical clinical presentations always warrant a thorough work-up, since cerebrovascular disease must be always considered [16]. As such, brain MRI is usually required to carefully screen for the underlying cause during the acute phase. Recently, transient T2*-weighted imaging abnormalities on brain MRI have been reported during the acute phase of MA [6-8]. The occurrence of these transient T2*-weighted imaging abnormalities after sclerotherapy lends support to the hypothesis whereby endothelin release and microembolization trigger CSD [17]. The two most likely explanations for these T2* findings relate to (i) increased oxygen consumption and a subsequent increase in the intravenous deoxyhaemoglobin concentration [18] and (ii) venous dilatation following the release of vasoactive factors (such as endothelin). In murine models, systemic levels of endothelin-1 (ET-1, one of the most potent vasoconstrictors and a CSD inducer) are significantly elevated one and five minutes after the initiation of foam sclerothapy [19]. Furthermore, Lemos et al.'s study of a population of Portuguese patients revealed a possible role for the endothelin receptor type A in migraine without aura [20]. Nevertheless, the prevalence and significance of these phenomena merit further investigation.

In conclusion, the present observation suggests that the transient nature of the T2*-weighted imaging abnormalities is associated with a CSD caused by migraine aura after sclerotherapy.

## Consent

Written informed consent was obtained from the patient for publication of this Case report and any accompanying images. A copy of the written consent is available for review by the Editor-in-Chief of this Journal.

## Competing interests

The authors declare that they have no competing interests.

## Authors’ contributions

YZ conceived the study, participated in its design and coordination and helped to draft the manuscript. MT has made substantial contributions to conception and design, acquisition of data, analysis and interpretation of data. MH has made substantial contributions to conception and design, acquisition of data, analysis and interpretation of data. DS participated in the design of the study, and helped to draft the manuscript. JMC has made substantial contributions to conception and design, acquisition of data, analysis and interpretation of data. JMB conceived the study, participated in its design and coordination, helped to draft the manuscript, and has been involved in revising it for important intellectual content. All the authors have participated sufficiently in the work to take public responsibility for appropriate portions of the content. All authors read and approved the final manuscript.

## References

[B1] Jia X Mowatt G Burr JM Cassar K Cook J Fraser C Systematic review of foam sclerotherapy for varicose veins BJS 2007 15 925 936 10.1002/bjs.5891 17636511

[B2] Guex JJ Allaert FA Gillet JL Chleir F Immediate and mid-term complications of sclerotherapy: report of a prospective multi-centric registry of 12,173 sclerotherapy sessions Dermatol Surg 2005 15 123 128 15762201 10.1111/j.1524-4725.2005.31030

[B3] Ratinahirana H Benigni JP Bousser MG Injection of polidocanol foam in varicose veins as a trigger for attacks of migraine with aura Cephalalgia 2003 15 850 885 10.1046/j.1468-2982.2003.00548.x 14510934

[B4] Coleridge SP Chronic venous disease treated by ultrasound guided foam sclerotherapy Eur J Vasc Endovasc Surg 2006 15 577 583 10.1016/j.ejvs.2006.04.033 16782367

[B5] Gillet JL Donnet A Lausecker M Guedes JM Guex JJ Lehmann P Pathophysiology of visual disturbances occurring after foam sclerotherapy Phlebology 2010 15 261 266 10.1258/phleb.2009.009068 20870875

[B6] Karaarslan E Ulus S Kürtüncü M Susceptibility-weighted imaging in migraine with aura Am J Neuroradiol 2011 15 e5 e7 20075095 10.3174/ajnr.A1973 PMC7964957

[B7] Shimoda Y Kudo K Kuroda S Zaitsu Y Fujinma N Terae S Sasaki M Houkin K Susceptibility weighted imaging and magnetic resonance angiography during migraine attack: a case report Magn Reson Med Sci 2011 15 49 52 10.2463/mrms.10.49 21441728

[B8] Bugnicourt JM Canaple S Lamy C Deramond H Godefroy O T2*-weighted findings in prolonged acute migraine aura Chin Med J 2013 15 20 24157179

[B9] Breu FX Guggenbichler S European consensus meeting on foam sclerotherapy Dermatol Surg 2004 15 709 717 10.1111/j.1524-4725.2004.30209.x 15099312

[B10] Kasner SE Chalela JA Luciano JM Cucchiara BL Raps EC McGarvey ML Conroy MB Localio AR Reliability and validity of estimating the NIH stroke scale score from medical records Stroke 1999 15 1534 1537 10.1161/01.STR.30.8.1534 10436096

[B11] Sarvananthan T Shepherd AC Willenberg T Davies AH Neurological complications of sclerotherapy for varicose veins J Vasc Surg 2012 15 243 251 10.1016/j.jvs.2011.05.093 21840152

[B12] Gillet JL Guedes JM Guex JJ Hamel-Desnos C Schadeck M Lauseker M Allaert FA Side effects and complications of foam sclerotherapy of the great and small saphenous veins: a controlled multicentre prospective study including 1025 patients Phlebology 2009 15 131 138 19470865 10.1258/phleb.2008.008063

[B13] International Headache Society Classification Subcommittee International classification of headache disorders, 2nd edition Cephalalgia 2004 15 suppl 1 1 160 10.1111/j.1468-2982.2003.00824.x 14979299

[B14] Viana M Sprenger T Andelova M Goadsby PJ The typical duration of migraine aura: a systematic review Cephalalgia 2013 15 483 490 10.1177/0333102413479834 23475294

[B15] Headache Classification Committee of the International Headache Society The international classification of headache disorders, 3rd edition (beta version) Cephalalgia 2013 15 629 808 23771276 10.1177/0333102413485658

[B16] Schoene J Sandor PS Headache with focal neurological signs or symptoms: a complicated differential diagnosis Lancet Neurol 2004 15 237 245 10.1016/S1474-4422(04)00709-4 15039036

[B17] Dreier JP Kleeberg J Petzold G Priller J Windmüller O Orzechowski HD Lindauer U Heinemann U Einhäupl KM Dirnagl U Endothelin-1 potently induces Lea˜o’s cortical spreading depression in vivo in the rat: a model for an endothelial trigger of migrainous aura? Brain 2002 15 102 112 10.1093/brain/awf007 11834596

[B18] Zagami AS Goadsby PJ Edvinsson L Stimulation of the superior sagittal sinus in the cat causes release of vasoactive peptides Neuropeptides 1990 15 69 75 10.1016/0143-4179(90)90114-E 2250767

[B19] Frullini A Felice F Burchielli S Di Stefano R High production of endothelin after foam sclerotherapy: a new pathogenetic hypothesis for neurological and visual disturbances after sclerotherapy Phlebology 2011 15 203 208 10.1258/phleb.2010.010029 21478144

[B20] Lemos C Neto JL Pereira-Monteiro J Mendonça D Barros J Sequeiros J Alonso I Sousa A A role for endothelin receptor type A in migraine without aura susceptibility? A study in Portuguese patients Eur J Neurol 2011 15 649 655 10.1111/j.1468-1331.2010.03239.x 20964792

